# Looking Back, Moving Forward: Challenges and Opportunities for Global Cervical Cancer Prevention and Control

**DOI:** 10.3390/v16091357

**Published:** 2024-08-25

**Authors:** Philip E. Castle

**Affiliations:** Divisions of Cancer Prevention and Cancer Epidemiology and Genetics, US National Cancer Institute, National Institutes of Health, 9609 Medical Center Dr., Room 5E410, Rockville, MD 20850, USA; philip.castle@nih.gov; Tel.: +1-(240)-276-7120; Fax: +1-(240)-276-7825

**Keywords:** Human papillomavirus (HPV), cervical cancer, HPV-related cancers, Pap testing, cytology, vaccination, gynecologic oncology

## Abstract

Despite the introduction of Pap testing for screening to prevent cervical cancer in the mid-20th century, cervical cancer remains a common cause of cancer-related mortality and morbidity globally. This is primarily due to differences in access to screening and care between low-income and high-income resource settings, resulting in cervical cancer being one of the cancers with the greatest health disparity. The discovery of human papillomavirus (HPV) as the near-obligate viral cause of cervical cancer can revolutionize how it can be prevented: HPV vaccination against infection for prophylaxis and HPV testing-based screening for the detection and treatment of cervical pre-cancers for interception. As a result of this progress, the World Health Organization has championed the elimination of cervical cancer as a global health problem. However, unless research, investments, and actions are taken to ensure equitable global access to these highly effective preventive interventions, there is a real threat to exacerbating the current health inequities in cervical cancer. In this review, the progress to date and the challenges and opportunities for fulfilling the potential of HPV-targeted prevention for global cervical cancer control are discussed.

**A.**  **Part 1: Looking Back**

## 1. Introduction

**Figure 1:**
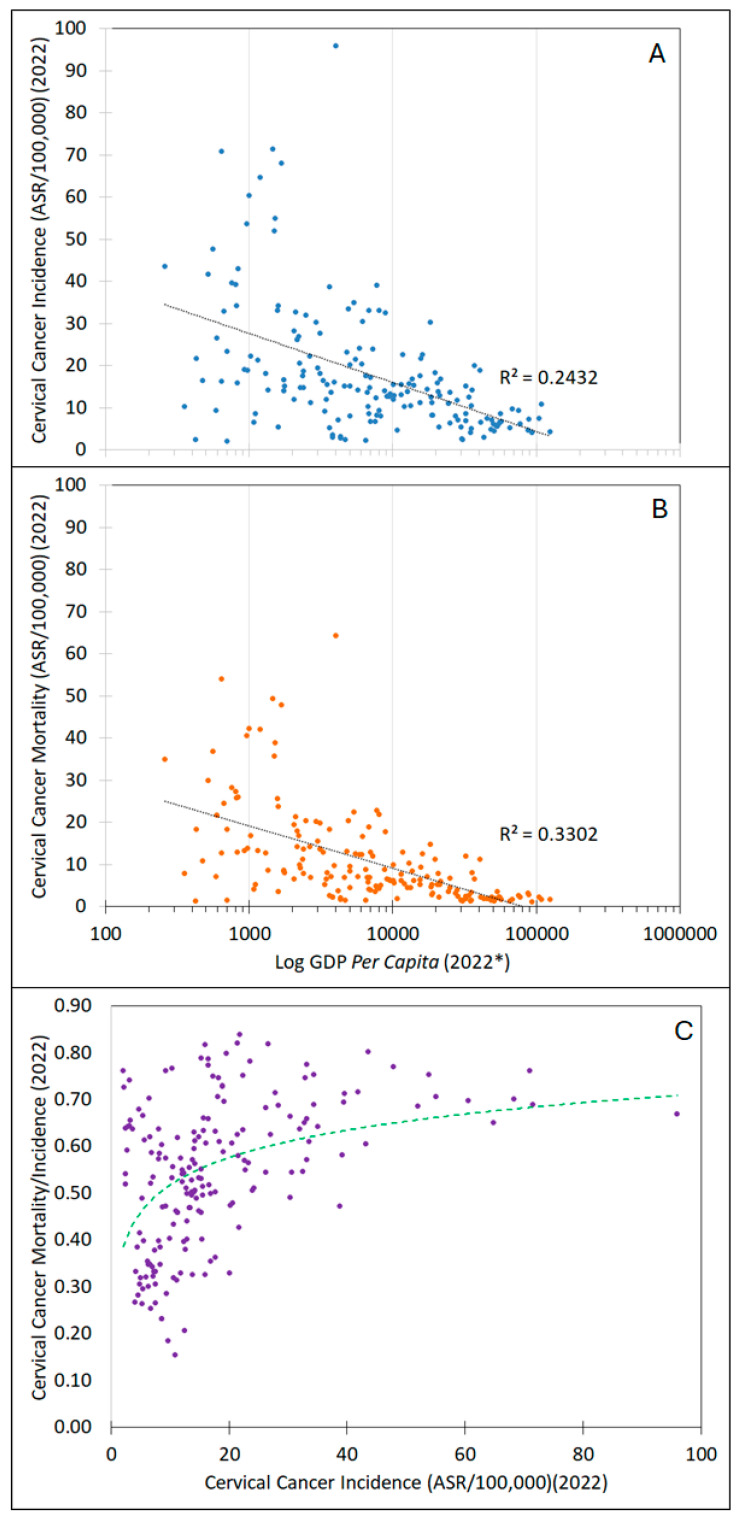
(**A**) country-specific relationship between 2022 cervical cancer incidence [1] and gross domestic product (GDP) *per capita* (on a log scale) in 2022 [11] (black dotted line shows the linear trend); (**B**) country-specific relationship between 2022 cervical cancer mortality [1] and GDP *per capita* in 2022 (black dotted line shows the linear trend); and (**C**) country-specific relationship between the ratio of cervical cancer mortality to incidence and 2022 cervical cancer incidence [1] in 2022 (green dashed line shows the logarithmic trend). * If GDP *per capita* was not available for 2022, the most recent data were used.

Cervical cancer is highly preventable. The implementation of widespread Pap testing, starting in the mid-20th century in high-income countries (HICs), led to significant declines in cervical cancer incidence, saving millions of lives. Unfortunately, these same benefits were not fully realized in underserved populations living in those same HICs and those living in low- and middle-income countries (LMICs). This was due to social inequities, geographical barriers, complexity, lack of quality control, and/or resource limitations, the latter of which include the lack of healthcare infrastructure, resources, and trained medical personnel to provide screening, i.e., testing and follow-up care. Consequently, cervical cancer became the “poster child” for cancer health disparities, with an order-of-magnitude difference in cervical cancer burden between the wealthiest and poorest countries [1] and significant differences between well-served and underserved populations in the wealthiest countries like the USA [2,3] and others [4,5,6,7,8,9,10]. Cervical cancer incidence and mortality were inversely correlated with gross domestic product *per capita*, and the ratio of cervical cancer mortality to incidence increased with greater the incidence (Figure 1).

It is now accepted that high-risk human papillomavirus (HPV) is the nearly obligate viral cause of cervical cancer [12]. This discovery catalyzed the development of two complementary, highly efficacious precision prevention strategies, i.e., those that directly target HPV including prophylactic HPV vaccination for primary prevention and HPV testing-based screening for secondary prevention. Despite these advances, more than two decades after their development and validation, cervical cancer remains a major cause of morbidity and mortality in women globally, with an estimated 662,000 new cases and 349,000 deaths attributed to cervical cancer in 2022, with the vast majority occurring in LMICs [1].

In 2018, the World Health Organization (WHO) announced a global call to action for the elimination of cervical cancer as a public health problem. In 2020, the World Health Assembly launched a Cervical Cancer Elimination Initiative [13]. Its strategy includes the following WHO “90-70-90” targets to be reached by 2030: 90% of girls fully HPV vaccinated by age 15 years, 70% of women screened twice using a high-performance test by the ages of 35 and 45 years, and 90% of women diagnosed with pre-cancer and 90% of women with invasive cancer treated. The “90-70-90” targets were designed to meet the “elimination” metric of incidence rates below four cases per 100,000 women. Of course, the target of treating 90% of invasive cancer will not contribute to the goal of reduced incidence but will benefit the population by reducing cervical cancer-related mortality and morbidity.

Together, these three interventions, cancer prophylaxis through HPV vaccination, cancer interception through HPV-based screening and treatment of cervical pre-cancers, and cancer mitigation through detection and treatment of early cancer, will have profound impacts on cervical cancer control, but each works on a different time horizon [14]. Mitigation will measurably benefit the population on the earliest time horizon through reduced mortality and morbidity, screening-based interception next, and finally, prophylaxis, which may take decades to observe its impact on cancer incidence. From modeling exercises, combining primary prevention through HPV vaccination and secondary prevention through HPV testing-based screening and treatment of pre-cancer and cancer will achieve the most rapid risk reduction towards achieving the WHO goal [15,16]. In order to accelerate mortality reductions and prepare for the anticipated sharp increase in screen-detected cancers needing treatment, should screening programs go to scale, mortality reduction targets also need to be set and included in the WHO’s goals.

At this juncture, as the field moves from basic and translational science to dissemination and implementation, it is a good time to review what has been achieved, why, and consider the challenges ahead to achieve global cervical cancer prevention and control. I provide my own perspective from my 25 years in the field. These lessons learned—and to be learned—can and should inform future efforts to control other cancers globally.

## 2. A Brief History

How did we arrive at this unique opportunity? The evolution of our understanding of cervical cancer is well described and discussed by others [17,18,19] and will be only recapitulated here briefly as it relates specifically to this discussion. It began with Hippocrates, at the beginning of the era of modern medicine and before the Common Era, who first documented and described HPV-related diseases of genital and skin warts, cervical precursors or lesions, and cervical cancer. He observed that cervical abnormalities, which he called “ulcers”, could progress to cervical cancer, providing the earliest insight into carcinogenesis [18]. Namely, cancer does not arise spontaneously but through intermediate, pre-cancerous changes that precede invasive cancer, which is still a foundational concept of carcinogenesis today.

Almost 2000 years later, 19th-century surgeon Domenico Antonio Rigoni-Stern made his famous observation about people engaged in sex work being more likely to die from cervical cancer than celibate nuns (the opposite for nulliparity and breast cancer deaths) [18,19], implicating a sexual factor in the development of cervical cancer. More than a century later, this observation fueled research to find the sexually transmitted causal factor, now known to be HPV.

In the early 1920s, physician George Papanicolaou first observed cancer cells in a cytology smear made from vaginal samples. Refinements in this method occurred over the next two decades [20], including improved staining [21] and sampling directly from the cervix [22], leading to what is known as the “Pap smear” for cervical cytology screening. Widespread adoption of annual Pap testing followed recommendations first in the mid-1940s, resulting in significant reductions in cervical cancer incidence and mortality in those places that could implement it effectively [23].

In the 1960s and 1970s, epidemiologic research hotly pursued the causal sexually transmitted infection, with a focus on herpes simplex virus. The discovery by Nobel-Laurate Harald zur Hausen and colleagues in the late 1970s into the early 1980s [24,25] of HPV genomes in cervical cancers revolutionized our understanding of the cause and the natural history of cervical cancer. Over the next decades, international, transdisciplinary teams of researchers, including but not limited to basic and clinical laboratory scientists, clinicians, epidemiologists and statisticians, public health scientists, and other disciplines, worked collaboratively to deepen our understanding of HPV and its role in cervical cancer as well as develop and validate prophylactic HPV vaccination and HPV testing-based screening, which are now seen as the standard of care for cervical cancer prevention and control worldwide [26,27].

## 3. HPV and Cervical Cancer

It is now understood that the sexual transmission and persistence of high-risk HPV genotypes (“types”) are the almost obligate cause of virtually all cervical cancer worldwide. Approximately 13 HPV types (HPV16, 18, 31, 33, 35, 39, 45, 51, 52, 56, 58, 59, and 68) have been designated as “high-risk”. In reality, there is a continuum of risk for HPV types. HPV16 and HPV18, the two high-risk HPV types targeted by first-generation HPV vaccines, cause approximately 70% of cervical cancer; HPV16 is the most carcinogenic HPV type, causing approximately 55–60% of cervical cancer, and HPV18 is the second most carcinogenic HPV type, causing approximately 10–15%. HPV31, HPV33, HPV 45, HPV52, and HPV58 together cause another 20% of cervical cancer. On the other end of the spectrum of high-risk HPV types, HPV68 is only considered a probable carcinogen, and the risk of cancer is considerably less than HPV16 [28,29,30]. Some HPV types, especially those phylogenetically related to the high-risk HPV types, can rarely cause cervical cancer [28,29,30,31,32]. This includes HPV66, which was mistakenly classified as high-risk [33] and now, unfortunately, is included in all second-generation HPV tests, providing very little benefit but reducing specificity, as it is commonly detected in low-grade abnormalities [32].

HPV infection also causes most cancers of the anus and a high percentage of cancers of the vagina, vulvar, penis, and oropharynx [34,35,36]. Almost 5% of cancers globally are attributable to HPV infection [36].

Other HPV types, notably HPV6 and HPV11, cause *condyloma acuminata* (genital warts) and recurrent respiratory papillomatosis. Still, other HPV types have no known link to human disease, and some appear to have some tissue tropism for vaginal epithelium [37,38,39,40], just as some non-genital HPV types have tropism for different skin locations [41]. Genital types unrelated to cervical cancer and not targeted by or protected from current HPV vaccines make for useful intrapersonal controls since they are all sexually transmitted concomitantly and may allow for single-arm, non-randomized HPV vaccine trials [42] by serving as markers for total HPV exposure.

The same 13 high-risk HPV types generally cause the same percentages of cervical cancer everywhere in the world [43] but with some notable variations in different racial/ethnic populations, such as higher percentages of HPV35-related cervical cancers in women of African descent [44,45,46] and HPV52-related cervical cancers in women of Asian descent [47,48,49], likely because of the evolutionary selection of viral variants within those populations. While HPV35 causes only about 2% of cervical cancer in the general population [28], it causes approximately 10% in women of African descent [44,45,46]. Although HPV35 is not currently included in prophylactic HPV vaccine formulations, hopefully, it will be included in future vaccines [50]. The relative public health importance of individual HPV types cannot be determined by their prevalence in the general population, or even in precursors to cancer, but must be determined in cancer itself.

HPV-negative cervical cancers do occur but are exceedingly rare once the denominator of cervical cancers is corrected for the HPV-related cervical cancers that are prevented (censored) by screening and treatment of HPV-related precursors. These HPV-negative cancers are pathologically defined as cervical but have molecular features that are shared with—and mostly resemble those of—endometrial cancer [51].

High-risk HPV prevalence is similar in exfoliated specimens from the vagina and the cervix [38,39,40]. However, despite the vagina having a much larger surface epithelial area to infect than the cervix (≤360 cm^2^ vs. ≤33 cm^2^, respectively [52,53]), cervical cancer is 20-fold more common cancer than vaginal cancer. That is, the cervix is at least 200-fold more susceptible to HPV-induced carcinogenesis than the vagina, which does not account for the vagina possibly being more exposed to HPV than the cervix, as the former is the first mucosal epithelium exposed to it. The physiological nature of the susceptibility to HPV-induced carcinogenesis of the annulus of tissue known as the cervical transformation zone, where cervical cancer primarily occurs, is not completely understood. However, a specialized stem cell, the cervical reserve cell, found under the columnar epithelium has been implicated as the susceptible cell for HPV transformation [54].

Cervical cancer develops along a simple, robust causal pathway with four reliably measured, natural history stages including the following: normal epithelium, hrHPV-infected epithelium, cervical pre-cancer (defined best as histologic diagnoses of cervical intraepithelial neoplasia CIN) grade 3 (CIN3) or adenocarcinoma in situ (AIS)), and invasive cancer [55]. HPV is the most common sexually transmitted infection. Indeed, among those who have ever been sexually active, it seems likely that nearly everyone has had an HPV infection, given that there are hundreds of HPV types and epidemiologic studies of incidence only detect a subset of those, infections can be acquired and cleared/controlled in the interval between observations, there are no observations over the entire sexually active life, and the entire lower genital tract can be infected but we typically only sample a small portion of it for HPV testing. In prospective cohorts, a high percentage of women test positive for HPV within a few years of observation, but the range of cumulative HPV positivity (approximately 30–80%) is wide, likely the result of differences in populations, the HPV test used, frequency of testing, and length of follow-up [56,57,58,59,60,61]. Natural history modeling estimated the lifetime probability of at least one HPV infection to be 85% for women and 91% for men [62], but, given the aforementioned factors, it could be higher still.

HPV persistence is the key determinant in the natural history of disease and is required for progression to pre-cancer and then invasive cancer [63,64,65]. However, not all persistent HPV infections develop into detectable CIN3/AIS, although it is probable that long-lasting, persistent HPV infection is essentially a “molecular” pre-cancer whether there is an accompanying histopathologic diagnosis of CIN3. CIN2 grade 2 (CIN2), the standard threshold of severity for treatment, is now understood to be an equivocal high-grade abnormality that is highly regressive, especially in younger women [66,67,68].

The transition probabilities between stages are difficult to observe directly, except for the acquisition of HPV infection, but they have been modeled [69]. The transition from HPV infection to CIN3/AIS is typically observed following prevalent HPV infection and, therefore, “left-censored”, i.e., the HPV infection has already persisted for some unknown amount of time. Likewise, the CIN3/AIS transition to invasive cancer is left-censored. The “Unfortunate Experiment” in New Zealand, in which treatment was purposely withheld from approximately 150 women with CIN3, found approximately 35% of CIN3 progressed to invasive cancer [70]. However, the median age of these women was 38 years, approximately 5–10 years after the peak of CIN3 in a screening cohort, and, therefore, their CIN3 was relatively “mature” [71]. The median time from HPV acquisition to cervical cancer detection ranges from 17.5 to 26 years, depending on the model used and its assumptions [69]. The age distribution of cervical cancer tracks with population behaviors and exposure to HPV, i.e., those populations that initiate sexual activity at a younger age tend to develop cervical cancer at younger ages, while those that start at an older age develop cervical cancer at older ages [72,73,74], although the effects of the latter may be muted by the effects of reduced circulating estrogen in peri- and post-menopausal women on hormonally responsive cervical tissue [72].

Condoms, when used correctly, are effective in blocking HPV transmission in women [75,76]. Male circumcision also reduces HPV carriage in men [77,78], and females with circumcised male partners are lower risk of getting HPV than those with uncircumcised male partners [77,78]. There is also some evidence that topical microbicides with carrageenan, a commonly used seaweed extract used as a thickening agent in food, provide broad-spectrum, safe protection against cervical HPV acquisition [79,80].

Despite 40 years of studying the natural history of HPV and cervical cancer and its clinical and molecular correlates, there are still important gaps in our knowledge. While we know that women who are immunosuppressed because of HIV or receiving a solid organ tissue transplant are at significantly higher risk of cervical cancer compared with immunocompetent women [81,82,83], the specific immunologic factors that play a role in determining the outcome of an HPV infection and clearance/control vs. persistence/progression in the general population are unknown. A better understanding of the immunological determinants of persistence would contribute to the development of effective HPV therapeutic vaccines, which, to date, have demonstrated effectiveness against CIN2/3 [84] that is much less than current standard-of-care physical (excisional or ablative) treatments [85]. A highly effective pan-HPV therapeutic vaccine or anti-viral, combined with HPV-based screening, could facilitate and accelerate global control of cervical cancer, especially in LMICs, where there is a huge gap in medical and health-system capacity to provide care and treatment to support the scale-up of screening [86].

There is also evidence of latency in HPV infections [87,88], which is a sub-clinical infection that is maintained and presumably controlled by the host immune system [87]. However, the clinical significance of latent HPV infections and how frequently they occur vs. true clearance remain unknown. A greater understanding of its role if any in cervical cancer diagnosed in older women would inform decisions about the age and other criteria for stopping screening.

## 4. Precision Prevention: Targeting HPV for Prophylaxis and Interception

A targeted, precision prevention approach of prophylactic HPV vaccination and HPV testing-based cervical cancer screening is now widely accepted as the new standard of care for the prevention of cervical cancer. The two strategies are complementary and combined can accelerate the control of cervical cancer [15] as follows: (1) HPV vaccination for long-term and perhaps lifetime cervical cancer risk reduction by preventing HPV acquisition and (2) HPV testing-based screening to detect and intercept pre-cancer through treatment before it becomes invasive to reduce cervical cancer risk immediately.

Prophylactic HPV vaccines produce high titers of HPV-neutralizing antibodies that block cervical HPV infection [89]. Antibody titers following HPV vaccination are an order of magnitude or greater than those that occur following natural infection, which provides partial protection against re-infection against the same type [90,91]. First-generation vaccines targeted HPV16 and HPV18; one product also targeted HPV6 and HPV11 to prevent genital warts and RRP. Different HPV vaccine formulations have been shown to provide different levels of cross-protection against untargeted but phylogenetically related high-risk HPV types [92,93,94]. A next-generation HPV vaccine includes additional five high-risk HPV types plus HPV16 and HPV18 [95]. A summary of HPV vaccines, the projected health benefits, and related biosimilars are shown in Table 1. Ignoring any future secular trends in HPV, it is reasonable to assume that the first- and second-generation of HPV vaccines could prevent approximately 3.5% and 4.5% of all cancers worldwide.

Registration clinical trials of HPV vaccines demonstrated nearly 100% reduction in cervical pre-cancer [89,100]. Recent reports from Finland, Denmark, Sweden, England, and Scotland have provided real-world evidence that HPV vaccination significantly reduces the incidence of cervical cancer [101,102,103,104,105]. HPV vaccination has been shown to be safe [106,107,108].

There is now evidence that protection against HPV endures for a decade or more with no evidence of waning immunity [109,110]. Importantly, there is a growing body of evidence that a single dose of an HPV vaccine is sufficient to provide long-lasting protection against targeted HPV types [110,111,112,113,114,115] and even cross-protection against untargeted but related types [92].

HPV vaccination of women living with HIV (WLWH) is well tolerated, safe, and generates adequate titers, albeit lower than populations without HIV [116,117,118]. However, there is currently no evidence that HPV vaccination is effective in protecting WLWH against HPV, cervical pre-cancer, or cancer [116,117,118].

HPV testing-based screening has replaced Pap testing/cervical cytology as the recommended method for secondary prevention of cervical cancer through the detection and treatment of cervical pre-cancer [26,119,120,121]. HPV testing is more sensitive but less specific for cervical pre-cancer and early cancer than cytology [121,122]. As a result of its greater sensitivity compared with other methods of screening, HPV testing more efficiently/effectively reduces cervical cancer incidence [123] and mortality [124], and a negative HPV test provides a more effective “rule-out”, i.e., reassurance against cancer [123,124,125,126], than cytology and other screening methods. The greater safety following a negative HPV test can be used to extend screening intervals, thereby reducing the harms of screening, or, in the same interval (as cytology), to reduce the risk further in screen-negative women. The U.S. Preventive Service Taskforce currently recommends routine quintennial HPV testing alone or concurrently with cervical cytology (“co-testing”) [119], although co-testing offers very little in terms of clinical performance above what HPV testing alone can [126].

Importantly, HPV testing-based screening permits the use of self-collection of cervicovaginal specimens or urine for HPV testing. In controlled research settings, HPV testing of self-collected cervicovaginal specimens performs comparably to provider-collected cervical specimens when a DNA amplification method of HPV testing is used [127,128,129].

Self-collection increases participation and acceptability of cervical cancer screening by not requiring an initial clinical visit for cervical screening and obviates the need for a pelvic exam with a speculum. Consistently, women prefer self-collected cervicovaginal specimens over provider-collected cervical specimens [130,131,132]. However, one of the primary barriers to the acceptance of self-collection is a lack of self-efficacy (i.e., women are concerned as to whether they can complete it “as well as the clinicians”), and women cite this as a reason that they might prefer provider-collected cervical specimens [130,131,132].

Urine collection for HPV testing obviates the need to insert a device vaginally and therefore may be more acceptable to some women, as well as some transgender men and nonbinary people with a cervix [133], in need of screening. Urine-based HPV testing may overcome cultural barriers to cervical cancer screening. Although there are few data for the HPV testing of urine, a similar comparability to provider-collected cervical specimens when a DNA amplification method of HPV testing is used has been shown [134]; however, further research is needed to optimize its use. Several studies report preference by women for, and greater confidence in the use of, urine over self-collected cervicovaginal specimens [135,136,137,138].

HPV-positive women can be triaged with a second, more specific test (also known as adjunctive or reflex testing) to “rule-in” those who need immediate further management (colposcopy and biopsy or treatment). The addition of secondary testing trades off immediate sensitivity for better specificity, with the goal of allowing some benign HPV infections to clear on their own. Thus, the population is stratified into low-risk (HPV-negative), who return to routine screening, intermediate-risk (HPV+/Triage−), who are followed at shorter intervals (increased surveillance), and high-risk (HPV+/Triage+), who need immediate care. Programmatic sensitivity and effectiveness depend on the sensitivity of the second test to identify immediately those who have cervical pre-cancer and cancer and losses in the follow-up of those who are HPV+/Triage−.

Cytology testing was the first method used as a triage test for HPV+, either performed concurrently (“co-testing”) or sequentially following an HPV+ result. Second-generation HPV tests have included some degree of HPV genotyping to identify those with HPV16 and HPV18 who are at the highest risk of cervical cancer [139,140,141] and warrant immediate clinical action or those with lower risk types that may be managed less aggressively/invasively [142,143,144].

New biomarkers have emerged that may replace or complement cytology and HPV genotyping as triage methods for the management of HPV-positive women. The most promising include p16/Ki-67 immunocytochemistry [145,146,147] and methylation of the HPV and host genomes [148,149,150]. p16/Ki-67 immunocytochemistry is now recommended for the management of HPV-positive women in the U.S. [145]. Molecular triage methods such as genome methylation might be particularly suited for self-collected specimens [151], which may not be collected in a way to preserve whole cells or have fewer diagnostic whole cells than found in a provider-collected specimen. New sequencing technologies that measure methylation without the need for bisulfite conversion may make methylation biomarkers more practical for clinical applications [152,153,154]. Another emerging approach is machine learning algorithm-based analyses of cervical images [155,156,157].

HPV E6 and E7 oncogene products remain an intriguing target for detection since low levels of E6 and E7 are necessary for genomic replication and amplification, but their overexpression is a biomarker for cervical pre-cancer and cancer [158,159]. Thus, a quantitative mRNA test with a low- and high-level cut point could function as both a screening and triage test, respectively. Detection of low levels, such as those achieved by commercial qualitative mRNA tests [160,161], would indicate the presence of HPV infection, and a negative result would rule out HPV infection, allowing for extended screening intervals. High levels of HPV E6/E7 mRNA are expected to have a high positive predictive value for cervical pre-cancer and cancer and should be more common among the more carcinogenic types, i.e., high levels of HPV16 E6/E7 mRNA should be much more common than HPV68 E6/E7 mRNA. An HPV16, HPV18, and HPV45 E6 oncoprotein test (now just HPV16 and HPV18) was shown to be less sensitive but highly specific for cervical pre-cancer compared with DNA detection [162], but this does not include a broad enough group of types to be clinically useful. It also needs further development so that it is scalable and reliable since it takes ~2.5 h to run a single test and is impacted by ambient testing conditions, respectively (personal observation).

However, the increasing number of tools for the prevention of cervical cancer may have the unintended consequence of increasing the complexity of providing care, leading to its suboptimal provision. To ensure appropriate care, the following strategies should be implemented: (1) risk-based decision-making to ensure equal care for equal risk [163] and (2) a risk calculator/decision support tool to guide providers [164,165].

Importantly, as discussed below, many of the barriers (access to healthcare, home and work obligations, financial toxicities, stigmatization, marginalization of/discrimination against racial, ethnic, sexual/gender minorities, etc.) to screening will impede follow-up care of HPV-positive women. These barriers must be addressed to realize the full benefits of adding self-collection, HPV testing, and other strategies to increase participation in routine cervical cancer screening.

## 5. Cervical Cancer: The Low-Hanging Fruit

It is worth noting that cervical cancer has some unique characteristics that make it uniquely preventable and controllable. Table 2 summarizes the following reasons why advances towards the prevention of cervical cancer have been exceptional: (1) a single etiologic agent, (2) slow development of cancer, (3) ease of tissue accessibility, (4) a small area of cancer susceptibility, and (5) a proven surrogate for cancer. While there are other cancers with one or more of these characteristics, no others have all five of these specific characteristics or are common enough that population-level interventions are warranted and potentially cost-effective.

Some of the key lessons learned from studies of cervical cancer include the following:

- Natural history informs interventions. The elucidation of the natural history of cervical cancer has guided the development and use of prevention strategies. It is now clear that younger women—and men—should be vaccinated against HPV before exposure to it but not screened because they have very little true pre-cancerous lesions and almost no cancer. However, mid-adult women need screening for long-persisting HPV infections that have developed into cervical pre-cancer and cancer but will benefit very little from HPV vaccination as they will acquire relatively few incident HPV infections that will go on to become cancer [166,167].
- Surrogates accelerate progress. Having a good surrogate of cancer allows for rapid cycling through novel interventions to identify those that are most promising without requiring an incidence or mortality endpoint. CIN3 and AIS have HPV genotype distributions that closely resemble that of invasive cervical cancer [144,168,169,170,171]; the positivity for biomarkers associated with cervical cancer increases with increasing certainty of pre-cancer [172,173]. Conversely, CIN2, which has been included in combined endpoint (CIN2 or more severe abnormality (“CIN2+”)) to help power prevention and screening trials, is highly regressive and is often caused by low-risk HPV, as a result of likely being an admixture of manifestations of HPV infection (e.g., CIN1) and CIN3 rather than a true biological entity [66,174,175,176]. Thus, its inclusion in a composite endpoint must be interpreted with caution. As a result of including CIN2 in endpoints, the clinical importance of certain HPV types can be overestimated. For example, HPV66, which commonly causes CIN2 and low-grade abnormalities [32], is included in all current HPV tests because of one influential study [33], despite the fact that it rarely causes cancer [28,29,32] and is not considered a high-risk HPV type [30,177]. Conversely, HPV18 is often under-represented in CIN3 compared with its prevalence in cervical cancer [32,168,170,178]. Thus, a weighted average based on histology and HPV genotype fraction based on cancer may better predict the impact of an intervention on cervical cancer risk than simple HPV type prevalence when a surrogate endpoint of CIN2+ is used [174].
- Screening works best as a two-step process. First rule out, then rule in. Typically, diagnostic tests are used to confirm a disease in a selected population enriched for the disease of interest, e.g., someone has a symptom, such as fever, and then a diagnostic test is run to determine the underlying cause. In the case of cancer screening, the intervention is performed in a population in which cancer and even precursors are relatively rare. In this scenario, a single-test screening algorithm will have poor positive predictive value (PPV) unless the test is extremely specific [179], which usually then sacrifices sensitivity. In the two-step, rule-out/rule-in algorithm, the first, more sensitive test (HPV) rules out disease in the healthy population. An important but underappreciated benefit of the rule-out algorithm is providing reassurance against disease, i.e., telling healthy people that they are healthy and at low risk of cancer. In the case of HPV testing, testing negative for the cause of cervical cancer allows screening intervals to be safely extended, reducing screening harms.
  When the triage test is applied to the sub-population of screen positives to risk stratify and determine who needs further evaluation (rule-in), the PPV is much better because the endpoint of interest is enriched [179]. Using a two-step, rule-out/rule-in algorithm, populations are stratified into three distinct risk groups, i.e., higher risk (rule-out+/rule-in+), intermediate risk (rule-out+/rule-in−), and lower risk (rule-out−), that can be managed according to clinical action thresholds. For example, HPV+/Pap+ women are sent to colposcopy, HPV+/Pap− are placed under active, annual surveillance (until there is evidence of increased risk in follow-up), and HPV− return to routine, 5-year screening. The results of the screening test can be combined with the triage test results for further risk stratification. For example, if HPV genotyping for HPV16 and HPV18 is available as part of HPV testing, the three tiers of risk are (1) HPV16+, HPV18+, or Pap+ go to colposcopy (rule-out+/rule-in+), (2) HPV+ but HPV16-, HPV18-, and Pap- undergo active annual surveillance (rule-out+/rule-in−), and (3) HPV- return to routine screening. As a consequence, more high-risk women are sent for immediate colposcopy, likely increasing programmatic sensitivity.
  Clinical action thresholds (CATs) can be established to guide the optimal management of women by maximizing the population benefits-to-harms ratio as well as promoting the principle of equal care for equal risk. CATs, based on risk, are used to guide clinical decision-making and are informed by sociocultural acceptance of tradeoffs in benefits and harms. Operationally, both biological (e.g., HPV16 detection) and non-biological risk factors (e.g., social determinants of health) are integrated into an individual risk estimate, and the CAT determines whether more (above the CAT) or less (below the CAT) aggressive intervention is warranted, e.g., colposcopy vs. surveillance, respectively.
- Implementing best practices is very difficult and slow. Even when the science and evidence are robust, adoption is slow, especially in “disorganized” healthcare systems, like cervical cancer screening in the U.S. It has been known that HPV testing is a better screening test than cytology for 20+ years, but, even now, very few women living in the U.S. get screened at the recommended screening intervals [180], screening tests are overused [181], and most U.S. cancer centers do not recommend HPV testing as the front-line cervical cancer screening test [182]. Vested interests almost certainly played a role in the slow change from cytology to HPV testing. Without HPV testing, self-collection will not be an option, which is key to reaching many women who cannot or will not undergo a pelvic exam or obtain care in the clinic. Implementation research on how to bring HPV testing into practice and de-implementing cytology-based screening and over-screening is greatly needed. By comparison, national, publicly funded healthcare programs, such as those in many European countries like The Netherlands, tend to be more efficient and have better adherence to guidelines, thereby reducing costs and harms of screening compared with the U.S. [183,184].
- Systematic Bias. The development of new technologies is subject to systemic biases. The HPV35 story is an example of such a bias. The formative epidemiological studies of cervical cancer did not include enough cases of cervical cancer in women of African descent to detect this important relationship and, consequently, HPV35 is not included in any current HPV vaccine formulations. Those studies that did include cases of cervical cancer from WLWH of African descent from sub-Saharan Africa and differences in type distribution were first attributed to HIV co-infection. Whether current multivalent HPV vaccines generate enough cross-protection to protect against untargeted HPV35 is unknown. However, it was recently announced that one vaccine manufacturer will develop a multivalent (> nine-valent) HPV vaccine that hopefully will include HPV35 [50].
- New technologies can exacerbate health disparities. The role of Pap in accelerating global cervical cancer prevention cannot be overstated. Millions of cervical cancers, and deaths due to cervical cancer, were averted worldwide because of Pap testing, though these benefits were concentrated in high-resource populations. Another important contribution of routine Pap testing was helping to elucidate the natural history of cervical cancer, which had profound consequences for the subsequent development of newer, more effective technologies directly targeting HPV. Having a Pap as a predicate test facilitated the development of HPV testing. In addition, Pap testing-based screening identified and validated CIN2/3 as precursors to cervical cancer, which allowed clinical trials to use them as an early, surrogate endpoint for invasive cervical cancer, which accelerated approvals of HPV testing and HPV vaccines. That said, it is time to sunset Pap/cytology for the next-generation test for screening, HPV testing, which, in addition to better sensitivity and negative predictive value, offers greater flexibility for screening through self-collection, allowing more women to get screened. Unfortunately, as discussed below, we are in danger of repeating the same mistakes as the wealthiest women are given preferential access to these new, more effective, HPV-targeted technologies for cervical cancer prevention.

## 6. Discussion

Despite the successes of Pap testing, the geographical variation in cervical cancer incidence and mortality in the U.S. and globally highlights important health inequities due to differential access to high-quality screening and follow-up care. In the U.S., the unequal burden of cervical cancer is related to many factors including but not limited to social, racial, sexual, and ethnic discrimination, low income/poverty, stigma, and geographic isolation such as rurality [3]. Communities with higher rates of cervical cancer mortality have poorer access to health care in general [185]. State- and county-level cervical cancer mortality is correlated with increased mortality of other preventable cancers, such as colorectal cancer, and they are inversely correlated with average *per capita* income [186]. Counties with persistent poverty (counties that have had at least 20% of their residents living below the federal poverty line continuously since 1980) experience significantly higher mortality rates in several cancers, including cervical, than those with non-persistent poverty [187].

Although HPV-based interventions have the potential to “close the gap” in the cervical cancer burden in the U.S., they also have the possibility of exacerbating it if the underlying causes of those disparities are not addressed [3]. The uptake of HPV vaccination, which was first approved by the FDA in 2006 and recommended by the Advisory Committee on Immunization Practices in 2007, has been unacceptably low. HPV vaccine coverage with at least one dose was 25.1% in 2007 and 54% in 2012 [188]. As of 2022, 78% and 65% of females aged 13–17 years received at least one and all recommended doses of HPV vaccine, respectively, which was similar to the coverage in 2021 (79% and 64%, respectively) [189]. The percentage of adolescents with at least one HPV vaccine dose declined in those insured by Medicaid and remained lowest among the uninsured [189]. By comparison, Australia achieved >70% coverage with three doses of the HPV vaccine in female children in its first year of introduction in 2007; differences across economic groups were significant [190] but less pronounced than in the U.S. By 2017, Australia achieved nearly 90% coverage for at least one dose and 80% coverage for three doses [191]. As of 2018, Australia had not yet reported a decline in the age-standardized annual rate of cervical cancer incidence [192].

There are several factors that have likely influenced the uptake of HPV vaccination in the U.S. and perhaps other places in the world. HPV vaccination is mandated only in Virginia, Hawaii, Rhode Island, and Washington D.C. By comparison, measles, mumps, and rubella vaccination and diphtheria, tetanus, and pertussis vaccination are mandated by all states and Washington D.C., and the coverage in 2021 was 91% and 80%, respectively. Hepatitis B vaccination, which requires three doses, is required by colleges and universities in about 30% of states and the coverage in 2021 was 91% [193,194]. So, while requiring vaccination undoubtedly improves coverage, its impact is somewhat variable.

Another factor that appears to influence HPV vaccine updates is the link between HPV and sex. Parents are still concerned that HPV vaccination may promote high-risk sexual behaviors in their children [195,196] despite the evidence that HPV vaccination does not promote compensatory sexual behaviors [197,198]. Messaging to parents and providers that de-emphasizes HPV vaccination as prevention for a sexually transmitted infection (STI) and emphasizing its cancer prevention benefits may help [199,200,201]. Delivering HPV vaccination as part of the early childhood vaccination schedule, as discussed in Part 2, would not only simplify HPV vaccine delivery but could help to distance it as an STI vaccine being delivered to preteens and early teens “before sex starts”.

The role of HPV vaccination in males warrants discussion. In general, gender-neutral (males and females) will prevent more cancers than female-only HPV vaccination. Importantly, men who have sex with men (MSM) may not benefit from the herd protection of HPV vaccination of females, as was demonstrated early following the introduction of HPV vaccination in women in Australia, where genital warts declined rapidly and sharply in women and heterosexual men but not MSMs [202]. However, while gender-neutral HPV vaccination is estimated to be a good value, it is generally less cost-effective than female-only HPV vaccination because females gain the majority of the benefits [203,204,205,206,207]. Thus, gender-neutral HPV vaccination might be a reasonable healthcare investment in HICs but rapid, high-coverage HPV vaccination in females should be the priority in LMICs.

**Figure 2:**
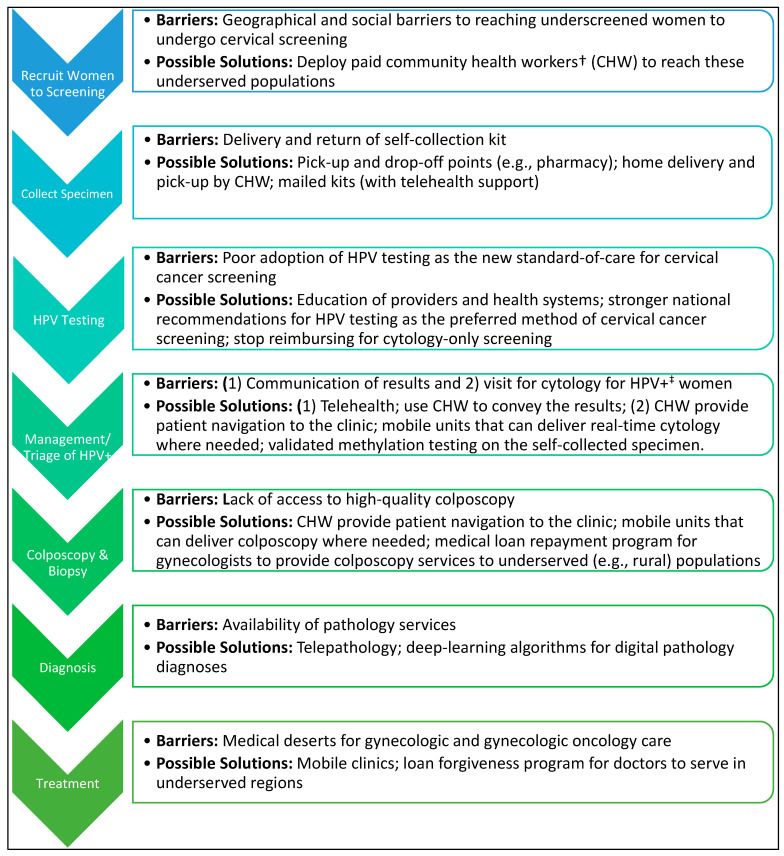
Some of the barriers and possible solutions for delivering self-collection and HPV testing for cervical cancer screening along the care continuum in the U.S. ^†^ Need to be paid members of the medical home, which will require a billable CMS code for their services. ^‡^ Women who test HPV positive (HPV+) but are negative for HPV16 and HPV18 will need an extra visit for cytology in the U.S. Self-collected specimens cannot be used for cytology because there are not enough diagnostic cells, and it is unlikely that the medium used for the self-collected specimen will preserve whole cells.

Expanding cervical screening in the U.S. via self-collection and HPV testing is a promising, complementary approach to reaching the sub-population of women who do not participate in current clinic-based programs. Yet, many of the underlying social determinants of health (SDoH)-related barriers to cervical screening remain [3] and will not be solved using self-collection alone. Figure 2 highlights some of those delivery barriers for self-collection-based cervical cancer screening. In the U.S. context, the optimal health service delivery model has not been established. Community health worker-based/door-to-door approaches to delivering self-collection devices consistently show greater increases in screening participation than more passive approaches (e.g., opt-out) [129,208] but they also require a greater commitment of resources. A recent trial in the U.S. among those receiving care at a U.S. integrated health care delivery system demonstrated a 17% increase in screening with mailed self-collection kits in women overdue for screening [209], but these women are not representative of those who are most underserved by the U.S. healthcare system.

There are obvious financial barriers to those who do not have insurance or have insurance but cannot afford the co-pay for any screening or follow-up care. Of note, recommended cervical cancer screening in the U.S. starts (age 21 years) and ends (age 65 years) largely before Medicare eligibility begins (age 62 years); therefore, other types of medical insurance coverage are necessary to pay for care. While the CDC’s National Breast and Cervical Cancer Program provides screening and diagnostic services [210], and treatment for cervical pre-cancer and cancer is made available to Medicaid-eligible women through state Medicaid programs under the Breast and Cervical Cancer Prevention and Treatment Act of 2000 [211], less than 7% of the 5.3 million eligible women accessed the program in 2017 [212]. Moreover, there are medical provider deserts, primarily in rural settings, of gynecologic and radiation oncologists to manage invasive cervical cancers [213,214,215] and gynecologists to biopsy and manage pre-cancerous abnormalities [216,217]. Because follow-up of screen-positives requires clinical visits and pelvic exams, these remain barriers to the completion of care.

In summary, highly effective tools to prevent cervical cancer are in hand. However, as discussed in Part 2, the hard work begins now, which is how to make these tools available to all.

**B.**  **Part 2: Moving Forward**

Although HPV-targeting interventions for global prevention and control of cervical cancer have been identified and are robust, their implementation presents many challenges. Most of these challenges are related to the lack of healthcare infrastructure and financial and human resources to implement them in LMICs. Investments to address these gaps lag far behind the technological advances and will be the bottleneck to achieving WHO goals to reduce the cervical cancer burden worldwide. Some of these barriers for each of the three target goals are discussed below and summarized in Table 3.

## 7. Achieving 90% HPV Vaccination

Prophylactic HPV vaccination is the ultimate cervical cancer risk reduction strategy, but it is not a panacea because there are several generations of adult women who will benefit little or not all from HPV vaccination. As a result, adult women generally will not be targeted by public health programs for HPV vaccination, especially those living in LMICs, because of the relatively poor cost-effectiveness compared with vaccinating younger females [219,220]. There has been some consideration of delivering HPV vaccination to adult women undergoing screening [221,222]. Such an approach would primarily reduce the endemicity of HPV in the population rather than prevent cervical cancer, since most HPV infections that ultimately cause cervical cancer are acquired before the age of 30 years [166]. It is also unknown whether multiple doses of HPV vaccine would be needed in this older population, which would logistically complicate its delivery. The cost and the cost-effectiveness of such an approach have yet to be determined and, given the significant barriers to delivering screening, this “faster HPV” approach may be limited to certain settings and target populations.

Relatively few women living in LMICs have received HPV vaccination compared with HICs [223], further exacerbating the cervical cancer health disparities rather than narrowing the gap. One-dose HPV vaccination should greatly increase its availability, deliverability, and affordability for LMICs. Yet, even with one-dose HPV vaccination, GAVI The Vaccine Alliance (“GAVI”) [224] subsidies, and Pan American Health Organization Revolving Fund discounted vaccine pricing [225], cost may still be a significant barrier, especially for those countries ineligible for support through these programs. Indeed, lower-middle-income countries, which are not eligible for GAVI support, have had much lower HPV vaccine uptake than low-income countries [223]. Strategies to increase the availability of lower-cost HPV vaccine doses, including access to biosimilars, expanding GAVI eligibility, and/or the development of a complementary global procurement mechanism, should help accelerate HPV vaccine coverage.

Perhaps a more significant challenge is the delivery of HPV vaccination to preteens and adolescents in some LMICs, especially those in sub-Saharan Africa (SSA), where health service delivery to this large segment of the population is typically limited and focused on reproductive health needs, although that is slowly changing [226,227,228,229,230,231,232]. Notably, as of 2020, 20% of the world’s population of adolescents, an estimated 250 million people, live in SSA, and that percentage of the world’s adolescent population is expected to grow to 25% by 2030 [233,234]. Thus, investment in the infrastructure, particularly in SSA, to develop more comprehensive adolescent healthcare, especially to deliver preventive services, could facilitate the delivery of HPV vaccination and have a broad impact on adolescent health. This could be school-based, although not all adolescents attend secondary education, and there is a significant decrease in school attendance with increasing age of adolescents, especially in low-income countries [235], so a secondary system may be needed to achieve high population coverage of HPV vaccination.

An alternative or complementary strategy is to deliver one-dose HPV vaccination, if proven safe and highly immunogenic, as part of routine infant/early pediatric (“early childhood”) vaccination, integrating it with other scheduled vaccines as part of WHO’s Expanded Programme on Immunization [236,237]. The approach is highly plausible. Many countries throughout the world have an early childhood immunization program, most of which have policies that recommend or mandate childhood vaccination, although vaccine coverage varies by country and correlates with country wealth [238,239,240,241,242]. Nevertheless, many countries have at least some capacity and infrastructure to deliver HPV vaccination to these young children. Moreover, existing data indicate long-term, age-specific anti-HPV titers are higher the younger a person is vaccinated [243,244,245], suggesting that the protective anti-HPV antibody titers could be even greater than those seen in preteens. Importantly, demonstrating that HPV vaccination delivered to infants and young children generates comparable immunity in adolescence to that generated by the current schedule in pre-adolescents would give vaccination programs more flexibility in the delivery of HPV vaccination at the age that is most convenient.

Those countries that have not introduced HPV vaccination then could choose the most convenient age group to deliver HPV vaccination for their context. For those countries in which adolescent HPV vaccination is already offered, two implementation strategies might be considered. The first is to continue vaccinating preteens and early adolescents while initiating HPV vaccination in infants and young children. When the first cohort of infants and young children reach the age of 9 years, vaccination of preteens is phased out. The second option is campaign-style outreach to vaccinate children from infant and early pediatric ages to the age at which female children are currently vaccinated, and then subsequently continue HPV vaccination only of infant/early pediatric females. Of course, other strategies might be considered and tailored to local needs and resources.

## 8. Achieving 70% HPV Testing-Based Screening

Globally, the varying ability to effectively implement Pap-based cervical cancer screening across the world has led to the current large disparities in cervical cancer burden. Pap testing under the best circumstances is only a moderately sensitive and reproducible screening method (compared with HPV testing) [122,246], and clinical performance can only be accomplished when robust, extensive quality assurance and control measures are taken [247]. There are now decades of experience demonstrating that Pap testing cannot be successfully and effectively implemented at a population level in LMICs [248,249,250,251,252], as indicated by the 10-fold disparities in cervical cancer burden worldwide [1], despite claims to the contrary [253,254]. Whether Pap testing is more affordable than HPV testing is irrelevant if it cannot be successfully implemented to scale in LMICs, and advocacy for it gives false hope and delays the implementation of technologies that can realistically reduce the burden of cervical cancer. In addition, the infrastructure, technical, and human capacities to provide Pap testing are specialized and cannot be adapted to address other healthcare needs in LMICs, thereby limiting the value of investing in it. Efforts should focus on bringing HPV testing technologies to everyone, rather than investing in Pap technology that cannot be scaled or sustained [253,255]. Or, as discussed below, resources should be devoted to other preventable/controllable diseases that burden the population even more than cervical cancer.

HPV testing is now considered the recommended method of cervical cancer screening [26,256] and, where available, should be considered the preferred, standard-of-care method. Modeling studies show that a program of HPV testing-based screening offers greater benefits and reduced harm compared with other methods in the general population of women and WLWH [257,258].

However, many countries do not have a recommended screening test and others still recommend cytology and/or visual inspection after acetic acid (VIA), not HPV testing [259]. Worldwide, most adult women have never been screened for cervical cancer with the following large differences in those who have ever been screened by any method by World Bank Economic Classification: 84% in high-income, 48% in upper-middle-income, 9% in lower-middle-income, and 11% in low-income countries [259]. As a first step towards achieving higher coverage for screening worldwide, national cancer control plans might consider including recommendations for cervical cancer screening, with HPV testing as the preferred method and a secondary method of choice as the stop-gap method until HPV testing becomes available and affordable.

Although HPV testing is more feasible than some methods and more effective than other methods for routine cervical cancer screening, there are formidable barriers to its global implementation, especially in LMICs. Importantly, screening is not a test but a multi-step process, the effectiveness of which is limited by the weakest step. Barriers in this process include the following: (1) the availability of LMIC-ready, robust, and effective HPV tests; (2) the limited number of gynecologists and clinicians who can provide colposcopic services and treatment of precursors; (3) the lack of pathology services; (4) weak health systems to identify, call, and recall women for screening and care, etc.; and (5) the lack of gynecologic oncologists to surgically remove early cancers and guide care and radiotherapy services to treat more advanced cervical cancers. A major limitation of the Cervical Cancer Elimination Initiative [13] is that it did not come with a plan to facilitate access to HPV testing and treatment.

Technical challenges for implementing HPV testing in LMICs have been discussed extensively and will be only briefly revisited here [260]. First, tests must have the necessary characteristics to perform well in many LMIC settings where there is only basic laboratory infrastructure and limited ability to run complex tests, and they must be performed under a wide range of environmental conditions. Second, these tests must be validated and easy to use, have a rapid sample-to-answer turnaround for some health service delivery models, and minimize biomedical waste, which many LMICs do not have the capacity to manage. Third, the testing cost, which includes not just the HPV test but all reagents, disposables, equipment (amortized over its lifespan), and personal protective equipment, must be low either because of the low cost of the technology itself or a global procurement strategy (e.g., a GAVI-like program or organization for in vitro diagnostics). Finally, the test must require very limited or no equipment; otherwise, all the technical issues related to equipment use and maintenance will be very problematic over time.

The ideal characteristics for an HPV test may differ by the care delivery model. Fundamentally, the three general delivery models include the following: (1) bring a test to a person; (2) bring a person to a test, i.e., a central facility that has testing capability; or (3) bring a specimen to a centralized testing laboratory.

In the first scenario, a single-use point-of-care (POC) test, such as a lateral flow test, might be well suited for a low-volume setting, whereas a small (e.g., 96-well) batch test on a clinical testing platform might be necessary for medium and higher volumes. In either case, testing will need to be performed in the most basic setting (e.g., rural health clinic), likely without any real laboratory infrastructure, and all testing (one or several tests) must be performed within an hour to minimize the time burden on the women being screened. In this setting, if it takes several hours to collect the necessary minimum number of specimens to run a batch test, or the batch test is run with fewer specimens, thereby increasing the testing costs, this approach becomes less attractive. Alternatively, campaign-style screening events (i.e., mobile screening units) could be performed with a batch test, but this would require the necessary logistics to move to different locations and provide comprehensive follow-up care.

In the second scenario, individuals come to a clinical facility to get tested and care on the same day. Like the first scenario, testing must be performed in short order to allow the completion of care in a timely manner, especially since women may travel significant distances/expend significant time coming to a facility, and there is a real risk of loss-to-follow-up if they are required to return for a second visit.

However, same-day screen-and-treat is challenging to implement at any scale, except in the context of research projects with dedicated personnel. In a real-world scenario, there are many barriers to same-day screen-and-treat, e.g., laboratory personnel have other tests to run and are not dedicated to HPV testing.

In the third scenario, specimens are collected remotely and tested centrally. Unlike the first two scenarios, rapid testing is not necessary since screening is not completed on the same day, but it would require linking the results back to the women and getting HPV-positive women back to a clinic for follow-up care. Some LMICs may have a specimen transport network, such as those used to transport blood for HIV and TB testing, which HPV testing specimens could leverage, as was the case with COVID-19 testing during the pandemic [261].

Screening algorithms usually rely on pathology to identify those at high risk, but that is not possible in many LMIC settings. In many LMICs, especially SSA, pathology services are very limited and unreliable, if available at all [262,263,264], and will not be able to handle any increased workload corresponding to scaled-up screening any time soon. Indeed, it is not uncommon to find stacks of unread cytology and histology slides in pathology labs throughout LMICs (personal observations). Machine-learning algorithms for the diagnosis of histopathology slides might provide a solution in the future [265,266,267,268]. However, many pathology labs do not have access to high-quality chemicals or equipment to fix and process biopsies, well-maintained equipment to section biopsies, or histotechnologists to prepare tissues. Therefore, scaled-up screening in these settings cannot rely on cytology as a triage test or biopsy for diagnosis to guide the management of HPV-positive women.

Non-pathology-based algorithms to manage HPV-positive women will trade programmatic sensitivity vs. specificity and pragmatic vs. accuracy. The most sensitive and simple algorithm is to treat all HPV-positive women immediately, but this leads to significant overtreatment by methods that may increase the risk of pre-term delivery [269]. Treating those only with the highest-risk HPV types such as HPV16, HPV18, and/or HPV45, which all next-generation HPV tests identify separately, would reduce overtreatment by roughly 70–80% while treating HPV types responsible for approximately 60–75% of cervical cancer. An alternative strategy that has been proposed is to screen with only the eight or so most carcinogenic HPV types that cause approximately 90% of cervical cancer, which is more specific than tests that include 13 or 14 HPV types. Speculatively, adding VIA to detect the highest-risk HPV types and find visually concerning abnormalities and cancer might incrementally increase the sensitivity of the triage step, but it may be subject to the same intra- and inter-provider variability that limits its effectiveness and at the “cost” of performing many more pelvic exams.

New technologies hold promise in identifying which HPV-positive women are at the highest risk of cervical cancer more effectively than VIA and could be combined with HPV genotype information to further risk stratification [155,157]. These methods also require a pelvic exam and so must be considered in the context of the aforementioned tradeoffs in performance vs. pragmatism. These include deep-learning/artificial intelligence optical image analysis of cervical images [155,157] and the use of optical fiber technology to provide an in situ, in vivo diagnosis [270,271]. Importantly, these technologies work in real time and thus, packaged with rapid sample-to-answer HPV testing, hold promise for one-visit, same-day screening algorithms, with only one pelvic exam and completion of care in under two hours. For remote specimen collection, an LMIC-ready host and/or viral methylation panel reflex test from the same HPV testing specimen could identify only the high-risk women who would need to come to the clinic to undergo a pelvic exam for treatment.

The issue of HPV test affordability is best addressed immediately through a global procurement strategy that buys HPV tests in large quantities to keep prices low as well as subsidizes costs to the end users, just as vaccines through GAVI [224] and other medications through The Global Fund. The currently available HPV vaccines would not be affordable otherwise; so, perhaps it should come as no surprise that neither are current HPV tests. In the future, lower-cost POC tests [272], near point-of-care batch tests [273], and high-throughput centralized testing will be more affordable and, therefore, sustainable, and the appropriate HPV-testing technology can be matched to the care delivery model. Still, these new HPV testing technologies will need an orchestrated procurement and subsidization to make them available, especially to those populations of women with the greatest need. Importantly, HPV testing implementation, especially using clinical testing platforms that are multi-analyte or adaptable to other diagnostic targets, will build important capacity in molecular diagnostics for other disease prevention, control, and management.

## 9. Achieving 90% Treatment

Regardless of the screening modality and delivery model, there must be a linkage to care to treat cervical cancer precursors and invasive cervical cancer. Otherwise, the full benefits of screening will be unrealized. Unfortunately, this component of the WHO strategic plan is the least developed, as it requires significant investment in building human and infrastructure capacities. Effective treatment of invasive cervical cancer requires human capacities in pathology, gynecology, gynecologic surgery/oncology, and radiotherapy [274,275]. To incentivize the investment and development of these capacities and infrastructure, the WHO cervical cancer elimination plan should include mortality reduction targets, e.g., reduce cervical cancer mortality by 50% by 2050, akin to President Biden’s aspirational and inspirational Cancer Moonshot goal of reducing all cancer mortality by 50% in 25 years [276].

Consider that there are approximately 8.1 billion people in the world now, if 7.5% of the world population are women aged 35–45 years, and only 10% of those have had even a single high-quality cervical screening in a lifetime, there are approximately 550 million women who need cervical cancer screening today. Assuming a 20% HPV prevalence and 0.2% cervical cancer prevalence, this will translate into an *additional* 110 million women in need of gynecologic care and 1.1 million women with screen-detected cancers that will need cancer therapeutic services.

There are few surgeons, anesthesiologists, and obstetricians in LMICs [277,278], which should be taken as a proxy for a lack of gynecology services for the treatment of cervical cancer precursors. There is also a lack of sufficient expertise to deliver tissue destructive (excision or ablation) treatment of cervical cancer precursors, which will result in both over- and under-treatment and sub-optimal effectiveness [279].

Few women with cervical cancer living in SSA receive the cancer care they need [280,281,282]. In fact, few African countries have the adequate human capacity, equipment, and supplies to treat cervical cancer. In a survey of African countries, where 43 of 57 responded and provided data, only 20% were deemed to have adequate gynecologic and radiation oncology staffing [283]. Twelve countries (22%) reported having no gynecologic oncologists while 24 of 31 countries (77%) with gynecologic oncologists had ≤ five gynecologic oncologists per 1000 cervical cancer cases [283]. Fourteen countries (26%) reported having no radiation oncologists, while 21 of 29 countries (72%) with radiation oncologists had ≤ five radiation oncologists per 1000 cervical cancer cases [283]. In comparison, for the approximately 14,000 cervical cancer cases diagnosed in the U.S. in 2023, there were approximately 1300 gynecologic oncologists (~93 per 1000 cervical cancer cases) and 5800 radiation oncologists (~412 per 1000 cervical cancer cases) [284]. A recent study of publicly available databases from 175 countries estimated that 57% of cervical cancers would require surgery, so an estimated 630,000 of the 1.1 million screen-detected cases would need surgery if screening was to scale up globally [215].

Effective treatment of advanced cervical cancer requires radiotherapy, which is best accomplished using a linear particle accelerator (LINAC) and brachytherapy. Yet, some LMICs do not have a LINAC, and those that do have insufficient numbers, often only one or two, to manage the total number of in-country cancer cases, including but not limited to cervical cancer, needing radiotherapy [280,283,285,286]. Indeed, the International Atomic Energy Agency ideally recommends four radiotherapy units per million people, with a minimum of at least 1.5 units per million, and most LMICs fall well short of that capacity [287,288,289]. In addition, when a LINAC is in disrepair, it might be years before it is repaired or replaced. Even when there is LINAC availability, there are also significant geographical and financial barriers to providing radiotherapy in many of these settings [280,290,291,292]. Likewise, brachytherapy is in short supply in Africa [283].

The situation in Uganda a few years ago provides an illustrative example. Starting in 2016, Uganda’s only LINAC, which provided the necessary radiotherapy for only 2.6% of those in need (1 LINAC vs. the minimum of 60 LINACs needed for a Uganda population of 39 million in 2016), was broken beyond repair, and Uganda had no in-country radiotherapy available [293]. Cobalt-60 radiotherapy was introduced in 2018–19 as a stopgap [294] until a single LINAC machine was available in 2021.

Several organizations [295,296,297] are working to address these gaps in cancer care by building human capacity and increasing access to radiotherapy. However, at the current scale of these admirable efforts, the demand is already well beyond the ability of these activities to address them, that is, before the substantial scale of screening in many countries.

Therefore, a concomitant increase in the capacity to treat pre-cancer and cancer in LMICs and low-resourced settings in HICs will need to accompany the scaling-up of HPV testing-based screening. However, while a commitment to increase treatment and diagnostic capacities is necessary, it will take years if not decades to achieve them, and alternative strategies should be considered in the interim. Neoadjuvant chemotherapy (NACT) followed by hysterectomy has been suggested as an alternative treatment regimen in the absence of standard-of-care cisplatin-based chemoradiation to treat locally advanced cervical cancer [290,298,299]. Yet, the evidence for the effectiveness of this alternative therapy is inconsistent and/or lacking [298,299,300,301,302,303]. Further research on NACT followed by hysterectomy is needed to establish its efficacy, for whom it works best, the best practices, and the training on how to implement it.

Is it ethically acceptable to scale up HPV testing and the treatment of pre-cancer without the concurrent capacity to treat invasive cancer? On one hand, it violates a well-accepted doctrine that care must be provided for all who are screened. Yet, many women would be spared from developing cervical cancer if there is sufficient capacity to treat pre-cancers even if most cancers could not be appropriately treated. The decision to introduce screening without the capacity to treat screen-detected invasive cervical cancer is an ethical dilemma, but the decision must be left to informed, in-country policy makers.

Although the WHO calls for two rounds of screening at ages 35 years and 45 years, perhaps it is worth considering only screening once in a lifetime for now and targeting women in a slightly younger and more narrow age group, e.g., 30–35 years of age, in whom there will be fewer screen-detected cancers and more but smaller pre-cancers that are more easily and effectively treated [304]. As noted, many countries have never had population screening for cervical cancer, and it will take time to build the human and infrastructure capacities to support it. Targeting a smaller population for whom it will be easier to provide care will give programs a greater chance at early success while building up the capacity to screen a larger population and treat more advanced disease.

There is a great need for effective HPV therapeutics, which could be coupled with HPV testing in a simplified screen-and-treat strategy. Unfortunately, there has been little success to date in developing a therapeutic HPV vaccine with efficacy against cervical pre-cancer that approaches that of current standards-of-care treatments (e.g., excision or ablation) (>80%) [84,305,306]. The efficacy of these experimental vaccines has been limited to approximately 20% and only against HPV16- and/or HPV18-related cervical pre-cancers, meaning that the population effectiveness for preventing invasive cancer is no more than ~15%. A study of topically applied artesunate showed approximately two-thirds of CIN2/3 regressed, but approximately half of those CIN2/3 retained the causal HPV infection, which means that it may not have cleared at all [307].

Notably, those trials included CIN2, which often regresses without treatment, especially in younger women [66]. Thus, even those additional CIN2 observed to regress during the trial period of observation in the intervention arm may be subject to time interval bias, and, had the cohort been observed sufficiently long, there would have been no difference in the regression between the intervention and control arms. In addition, CIN2 is an admixture of CIN1 and CIN3, i.e., either misclassified CIN1 or CIN3, and a CIN2 diagnosis is poorly reproducible [176,308], so the transition from CIN2 to CIN1 may not be truly regression but reclassification. Thus, the population effectiveness in reducing cervical cancer risk of these HPV therapeutics may be significantly overestimated.

A recent meeting was convened to describe a preferred product profile for an HPV therapeutic vaccine [86]. Local priming at the cervix may be necessary to recruit effector T-cells across the basement membrane of the cervical epithelium to the site of the abnormality [309]. However, as noted, an HPV therapeutic vaccine may be less effective in WLWH, in whom 6% of cervical cancer occurs [310], because they are immunocompromised. Thus, a complementary strategy to develop a non-immune-related biological against HPV, such as an antiviral, should be considered.

More fundamentally, what is the minimum acceptable effectiveness of such biological agents as a substitute for standard-of-care tissue-destructive treatments? The aforementioned meeting [86] suggested that 50% direct efficacy against targeted HPV16- and HPV18-related CIN2/3 and 50% cross-efficacy against CIN2/3-related types is the minimum. By inference, this would suggest an efficacy of 50%, but that assumes equal efficacy against CIN3 and CIN2, the latter of which is more likely to regress on its own [66], and that women get the full regimen of multiple treatments. Nor is cross-efficacy assured, at least for a therapeutic vaccine, given that they typically target E proteins that are less well conserved between types than L proteins, which do show some cross-protection as prophylactic vaccines, but not so much that it stopped the development of next-generation prophylactic vaccines are multivalent to provide broader protection. Thus, it is reasonable to assume that effectiveness might be significantly less than 50% for the base-case product. When used correctly, tissue-destructive methods, such as excision and ablation, are highly effective (~5–10% failure rate, i.e., 90–95% effective) [311], but, as discussed, they are much more difficult and more resource-intensive to deliver effectively. Is it ethical to deliver the lesser therapeutic option knowingly, given that it is likely to be less efficacious but potentially more effective? Ultimately, should such a biological therapeutic emerge with lower efficacy, local policy makers will need to weigh those tradeoffs and decide.

## 10. Cancer Care

Although not included in the WHO’s targets for cervical cancer control, palliative care is a critical component in the cancer care continuum and cannot be overlooked. Women living in LMICs and identified clinically or by screening with incurable late-stage cervical cancer will need palliation for the highest quality of life for as long as possible. However, like with the other components of a comprehensive cervical cancer control program, there are huge health inequities between HICs and LMICs in terms of access to palliative care and opioid medications for pain control and cancer care [218,312,313,314,315]. As of 2013, no African country had all seven essential opioid formulations (immediate-release oral morphine; controlled-release oral morphine; injectable morphine; oral immediate-release oxycodone; transdermal fentanyl; oral methadone) recommended by the International Association for Hospice and Palliative Care [218]. A number of factors impact access to these medications, including eligibility restriction, physician prescriber restrictions, no emergency prescriptions by fax/phone or non-medical prescribing, limited prescription duration, no pharmacist authority to correct prescription, and increased bureaucratic burden of prescriptions, restricted dispensing sites, and negative language in laws.

## 11. Other Barriers

Another very underdeveloped capacity for delivering a comprehensive cervical cancer elimination plan is the maintenance and repair of equipment. As noted, LINAC machines fall into disrepair, and there must be a plan in place to maintain them [316], especially in the lowest-resourced countries in the world, where LINAC availability is already well below what is needed. Human and infrastructure capacities to provide preventive maintenance and repairs for equipment are greatly lacking in LMICs [317]. It is very common to walk through clinics and hospitals and see hallways cluttered with broken donated state-of-the-art equipment that may never be repaired (personal observation) because often the equipment needs to be shipped to another continent, usually the U.S., Europe, or Australia, for repair and that just does not happen. Thus, the cost is more than the equipment itself, and investment in the infrastructure to provide maintenance and repair services locally will be critical for the sustainability of a cervical cancer control program.

## 12. Other Opportunities for Global Cancer Control

It is expected that other HPV-related cancers, anogenital cancers (anus, vulva, and vagina), and oropharyngeal cancer will decrease significantly from HPV vaccination, but, like with the cervix, it will take years if not decades before the impact will be observed. Strategies to pool data from countries that have adopted HPV vaccination early and have good cancer registries (preferably with medical record linkage to vaccine status) will help accelerate the generation of evidence that HPV vaccination is a broadly protective, cancer-preventive vaccine and hopefully encourage its greater uptake.

Treatment of anal cancer precursors reduces anal cancer incidence [318]. There are recommendations for screening, by cytology and/or HPV testing, and management of positives, primarily targeting high-risk, HIV-infected individuals (men who have sex with men and transgender women) but also extending to intermediate-risk individuals [319]. Given the greater morbidity in treating precursors of anal cancer compared with cervical cancer and the lack of providers who can provide high-quality anoscopy, restricting to high-risk individuals is the most practical and likely to be the most cost-effective.

Targeting other oncogenic infections, such as Epstein–Barr virus (EBV), which causes nasopharyngeal cancer and lymphomas, hepatitis C virus (HCV), which causes liver cancer, and *h. pylori*, which causes non-cardia gastric cancer, is perhaps the next best opportunity to prevent/control a significant number of cancers globally. Active HCV can be detected using mRNA assays and treated with directly acting anti-viral medications to prevent liver cancer, but only 20% of liver cancers are due to HCV [320]. Nevertheless, a vaccine against HCV could simplify its global control. EBV vaccines are in development [321,322], but there is no identified nasopharyngeal precursor, and sampling the nasopharynx is more invasive than sampling the cervix. Whether a vaccine demonstrating that it prevents or treats EBV, or even prevents multiple sclerosis [323], is sufficient for regulatory approval instead of a cancer endpoint is unknown. Screening for EBV serum biomarkers for NPC control in high-risk populations is promising [324].

Gastric cancer [325] is one of the most common cancers, with an annual incidence of 1.1 million cases and an annual mortality of 0.77 million people, globally. Like cervical cancer, gastric cancer is characterized by order-of-magnitude differences in burden between high-burden and low-burden countries, like the U.S. Most of those gastric cases are non-cardia cancers caused by *H. pylori* infection. In the U.S., gastric cancer is a multiple disease with multiple causes, with *H. pylori*-related non-cardia cancer mostly affecting immigrant populations from high gastric cancer-burden countries [326,327,328]. Although population-level antibiotic treatment of *H. pylori* infection significantly reduces the carriage of *H. pylori* [329], *H. pylori* infection recurs soon after antibiotics are stopped [330], and the widespread use of antibiotics raises concerns about antibiotic stewardship in general and antibiotic resistance of *H. pylori* [331,332,333,334]. Yet, despite the overall global burden of and large health disparities in gastric cancer, research on developing alternative strategies for gastric cancer prevention and control is lagging [335]. Like with cervical cancer, a multi-prong strategy of targeted prophylaxis to prevent or treat early *H. pylori* infection, screening and interception of chronic *H. pylori* infection or possibly gastritis [336,337,338], and mitigation through early detection of gastric by endoscopy of high-risk populations might be considered now, but a biological (e.g., vaccine) against *H. pylori* would greatly facilitate gastric cancer control [339,340,341]. Novel delivery strategies for screening might improve coverage and cost-effectiveness, e.g., combining fecal screening for *H. pylori* antigen or DNA and colorectal cancer testing (FIT) to increase screening for both gastric and colorectal cancer [342,343,344], and targeting families of those known to have a *H. pylori* infection, since they likely share the same *H. pylori* infection, for screening and *H. pylori* treatment [345]. If an *H. pylori* vaccine is therapeutic, an *H. pylori* screen-and-vaccinate approach might be highly effective.

## 13. Final Comments

Cervical cancer is the cancer for which we have the greatest opportunity, through HPV-targeted interventions, to control and reduce the burden of a cancer worldwide. As discussed, there are several advancements that would accelerate this process, which are highlighted in Table 3. Still, even with current technologies, we could save millions of lives over the next decades.

Indeed, if we cannot do it for this cancer, what chance do we have to do it for any other cancer? Unfortunately, those with greater resources are the ones who are given preferential access to newer, more effective, HPV-targeted technologies, rather than equitable access for all, potentially exacerbating health inequities first introduced with Pap testing. We are now challenged to reverse the historical trends for these and virtually all health technologies and interventions and achieve universal access and delivery to “close the gap”.

A comprehensive care program, from prophylaxis to palliation, must include the missing but necessary investments to build human and healthcare infrastructure capacities (including electronic health records) for delivery, as well as a global procurement strategy that makes access to these life-saving and live-improving interventions equitable. While the WHO’s call to action for cervical cancer elimination as a public health problem is inspirational, a major human- and infrastructure-capacity-building investment must be made to realize it. Who is going to make that commitment?

The question of “How good is good enough?” also needs to be asked in relation to cervical cancer control. If the world’s female population is vaccinated with a multivalent HPV vaccine at sufficiently high coverage, the incidence and mortality of cervical cancer will likely decrease almost an order of magnitude compared with today’s rates and approach the age-standardized rate of 4 cervical cancers per 100,000 that WHO has set out as threshold for cervical cancer elimination as a public health problem. Although a combined strategy of HPV-based screening with HPV vaccination will accelerate the control of cervical cancer compared with HPV vaccination alone [15], its implementation is much more challenging because of the greater costs and human resource and logistical requirements, and, on a population level, fewer benefits and more harms. HPV vaccination is being implemented more rapidly than HPV testing because vaccination is a “simpler” process, i.e., one shot and one visit, vaccines are cheaper than HPV tests, and nearly every country has some infrastructure and expertise for vaccination; most if not all of the 194 WHO member states have at least diphtheria–tetanus–pertussis, hepatitis B, *Haemophilus influenzae* type b, measles-containing, and polio vaccines, and more than 60% have HPV vaccines, included in their routine vaccination schedules [346]. Several countries in Latin America started vaccination more than a decade ago, and they are still struggling to implement HPV screen-and-treat. In the absence of those investments needed for screening, interventions that may be best buys and the easiest to implement might be those at the beginning and end of life through prophylactic HPV vaccination in young childhood and palliation, including access to narcotic drugs for pain control, for incurable cervical cancer, respectively.

Unfortunately, the roll-out of HPV vaccination in LMICs has been much slower than desirable, and relatively few women living in LMICs have received HPV vaccination since it was first available almost two decades ago. As a consequence, there was a missed opportunity to HPV vaccinate approximately 1 billion preteen women over the last ~15 years, which could have averted approximately 10 million cases of cervical cancer over the next 30 years.

Importantly, while cervical cancer is uniquely preventable, its prevention must be placed in the context of local needs and resources: it is not a leading cause of death worldwide or in any World Bank classification of economies or continents [347]. Meanwhile, other preventable causes of death, such as heart disease and diabetes, are far more common causes of mortality [347,348]. Lack of access to clean water and associated diarrheal diseases kills more than an estimated 1.2 million people worldwide, many of whom are children living in LMICs [347,349]. Therefore, local needs and priorities should drive resource allocation to cervical cancer programs.

An investment in cervical cancer prevention and control could be catalytic if made with an eye toward addressing the broad set of health needs and inequities experienced by resource-constrained populations. As noted, the high cervical cancer burden in regions within HICs and LMICs across the world is a signpost for cancer and other health disparities [185]. We need to consider women as a whole being, not just their cervix, and their family and community if we are to move towards health equity globally. Reducing maternal mortality, e.g., cervical cancer-related mortality, reduces intergenerational consequences of those deaths, including orphaning of children and childhood death [350,351].

Such an investment would build the human, health system, and technical capacities to address the cluster of chronic and non-communicable diseases that still differentially burden lower-resourced populations [186]. This includes improved health systems and the introduction of electronic health records [352] to track and guide the care of patients, human capacity in medicine and public health to deliver care, molecular diagnostics, palliative care, etc. Just as the initiation of the 20th-century space program undoubtedly led to the technological revolution in the 21st century, an investment in cervical cancer prevention and control in the 21st century could lead to a health revolution in LMICs and HICs in the 22nd century. The World Health Organization (WHO) has a call to action. We have talked the talk—will we walk the walk?

## Figures and Tables

**Table 1:** Overview of HPV vaccines.

	Quadrivalent HPV Vaccines	Bivalent HPV Vaccines	Nonavalent HPV Vaccines
U.S. FDA-approved product name	Gardasil	Cervarix	Gardasil-9
Manufacturer	Merck	GSK	Merck
Virus-like particle type and dosing	40 mg HPV16; 20 mg HPV18; 20 mg HPV6; 40 mg HPV11	20 mg HPV16; 20 mg HPV18	60 mg HPV16; 40 mg HPV18; 30 mg HPV6; 40 mg HPV11; 20 mg HPV31; 20 mg HPV33; 20 mg HPV45; 20 mg HPV52; 20 mg HPV58
Adjuvant	225 mg amorphous aluminum hydroxyphosphate sulfate	500 mg aluminum hydroxide and 50 mg 3-O-desacyl-4′ monophosphoryl lipid A (MPL)	500 mg amorphous aluminum hydroxyphosphate sulfate
Projected, estimated prevention benefits	70% of cervical cancers; 90% of warts	84% of cervical cancers ^†^	90% of cervical cancers; 90% of warts
Biosimilars	Cervivac ™ [96] (Serum Institute of India, Pune, India)	Cecolin ^®^ [97,98] (Xiamen Innovax Biotech Co. Ltd.; Xiamen, China); Walrinvax (Walvax Biotechnology Co.; Yunnan, China)	Cecolin 9 ^®^ [99] (Xiamen Innovax Biotech Co. Ltd.; Xiamen, China)

^†^ Assuming cross-protection against untargeted HPV types [92,93,94].

**Table 2:** Characteristics that make cervical cancer uniquely preventable.

Characteristic	Comment
A. Single etiologic agent	Approximately 13 HPV types cause virtually all cervical cancer worldwide. There are no other cancers for which there is a single, identifiable causal agent.
B. Slow-growing cancer	Average sojourn time from HPV exposure (initiation) to cancer is ~25 years.
C. Tissue accessibility	The cervix can be sampled directly by a brush for cytology and molecular testing for screening and by biopsy forceps to collect tissue for diagnosis with an outpatient speculum exam. The relative acceptability of sampling from this tissue allowed the early development of Pap testing, which was key to elucidating the natural history of cervical cancer, including the identification of a good surrogate (see D). Cervicovaginal sampling collects sufficient amounts of HPV for detection that self-collection is feasible.
D. Small area of susceptibility	The vast majority of HPV-related genital cancers occur in a very small annulus of tissue, the cervical transformation zone, with the most distal (from the vaginal opening) boundary defined by the squamocolumnar junction, which can be visualized, making sampling and diagnostic biopsies much simpler.
E. Proven surrogate for cancer	CIN3/AIS have some characteristics of invasive cervical cancer, most notably an HPV-type distribution. A proven surrogate permitted the more rapid validation of novel, HPV-targeted intervention strategies including HPV vaccination and HPV testing-based screening.

**Table 3:** Some of the challenges and potential solutions to implementing WHO interventional targets to achieve cervical cancer elimination. Bold type highlights areas in need of additional research.

**HPV Vaccination**		**Challenge**	**Potential Solution (s)**
	Financial	Cost of vaccines.	One-dose HPV vaccination. Expand GAVI eligibility and include Gardasil-9. Use lower cost biosimilars.
	Technical/Logistical	Lack of adolescent health platform.	Develop adolescent health platform. **Include HPV vaccination of infants WHO EPI vaccine schedule.**
	Human Capacity	Expand the providers who can provide HPV vaccination.	Training and certification of community health workers to vaccinate.
	Infrastructure	Cold chain for vaccine delivery.	Develop cold-chain infrastructure. Develop temperature-resistant VLP vaccine.
**HPV Testing-Based Screening**		**Challenge**	**Potential Solution(s)**
	Financial	Test cost.	Development of low-cost testing technology. Establish a global procurement strategy for laboratory tests and testing.
		Cost of disposables (pipet tips, PPE, etc.).	Tests that require minimal specimen handling and processing. Global procurement strategy for laboratory tests and testing.
	Technical/logistical	Getting the specimen to the HPV test.	Development and validation point-of-care or near point-of-care HPV tests. Use pre-existing or develop specimen courier networks to transport specimens to central testing laboratory.
		Management of HPV-positive women.	**Develop and validate deep learning algorithms for image analysis to distinguish those with and without cervical pre-cancer.** **Develop a robust methylation assay that works from a self-collected specimen.**
	Human capacity	Number of trained technicians,	Expand training and retention of laboratory technicians; develop assays that require minimal training.
	Infrastructure	Lack of qualified labs with “clean rooms” for PCR-based testing.	Develop assays that do not require PCR safe testing environments.
	Other		
**Management/Treatment of Precursors**		**Challenge**	**Potential Solution (s)**
	Human capacity	Limited capacity for gynecologic services including colposcopy and treatment of precursors.	Increase gynecology training. **Develop pan-HPV Therapeutics**
		Limited capacity for pathology.	Screen and treat; screen, triage (non-pathology methods), and treat. **Develop an AI-based digital pathology platform.** Increase human capacity in histotechnology and pathology.
	Infrastructure	Lack of clinics to provide services.	Mobile clinics.
**Cancer Treatment, Management, and Care**			
	Financial	Cost of treatment.	Subsidized care based on ability to pay.
	Technical/logistical	Long distances to reach health care facilities with cancer care capacities.	Dedicated transportation for cancer care.
	Human capacity	Lack of gynecologic oncologists. Lack of radiation oncologists and technologists. Lack of pathologists.	Training and mentoring programs for staffing of gynecologic oncology, radiation oncology, and pathology services.
	Infrastructure	Lack of LINACs. Lack of brachytherapy.	Place more LINACs. Increase availability of brachytherapy. Validate the use of neoadjuvant therapy and surgery.
	Policy	Access to and acceptance of morphine/opioids for pain management [218].	Policy changes and de-regulation of morphine/opioids. Education and training on their use and abuse.
